# Humanin enhances the cellular response to stress by activation of chaperone-mediated autophagy

**DOI:** 10.18632/oncotarget.24396

**Published:** 2018-02-03

**Authors:** Zhenwei Gong, Inmaculada Tasset

**Affiliations:** Inmaculada Tasset: Department of Developmental and Molecular Biology, Institute for Aging Studies, Albert Einstein College of Medicine, Bronx, NY, USA

**Keywords:** oxidative stress, chaperone mediated-autophagy, proteostasis

Increased oxidative stress and loss of proteostasis are characteristics of aging. Failure to remove the oxidative stress-damaged components has been recognized to play critical roles in the pathophysiology of common age-related disorders including neurodegenerative disease such as Parkinson’s disease and Alzheimer’s disease, and cardiovascular diseases such as myocardial infarction and heart failure. Strategies to diminish oxidative stress or effectively eliminate oxidative-damaged intracellular proteins may therefore provide novel therapeutic option for many age-related diseases.

Chaperone-mediated autophagy (CMA) allows for selective degradation of soluble proteins in lysosomes, contributing to the cellular quality control and maintenance of cellular energetic balance [1]. CMA substrate proteins are targeted by the chaperone hsc70 to the lysosomal surface where, upon binding to the lysosome-associated membrane protein type 2A (LAMP-2A), they are translocated into the lysosomal lumen for degradation. CMA is activated by oxidative stress to facilitate degradation of damaged proteins [2], thereby eliminating the insults of oxidative stress. Given the fact that CMA activity declines with age [3], and oxidative damage in cells increases during aging, CMA activators hold the potential for development as a new generation of treatment option for age-related diseases.

In our recent study, we identified that humanin (HN), an antiapoptotic, mitochondria-associated peptide is an endogenous CMA activator [4]. We demonstrated that HN protects multiple cell types including cardiomyoblasts, primary cardiomyocytes and dopaminergic neuronal cells from oxidative stress-induced cell death in a CMA-dependent manner. In fact, this protective effect is lost in CMA-incompetent cells (LAMP-2A knockdown). Both exogenously added HN as well as the endogenously generated HN cooperate in CMA activation. Thus, knockdown of endogenous HN decreases CMA activation in response to oxidative stress. Both endogenous and exogenous HN localize at the lysosomal membrane where they cooperate to enhance CMA efficiency. HN acts by stabilizing binding of the chaperone HSP90 to the upcoming substrates at the cytosolic side of lysosomal membrane.

**Figure 1:**
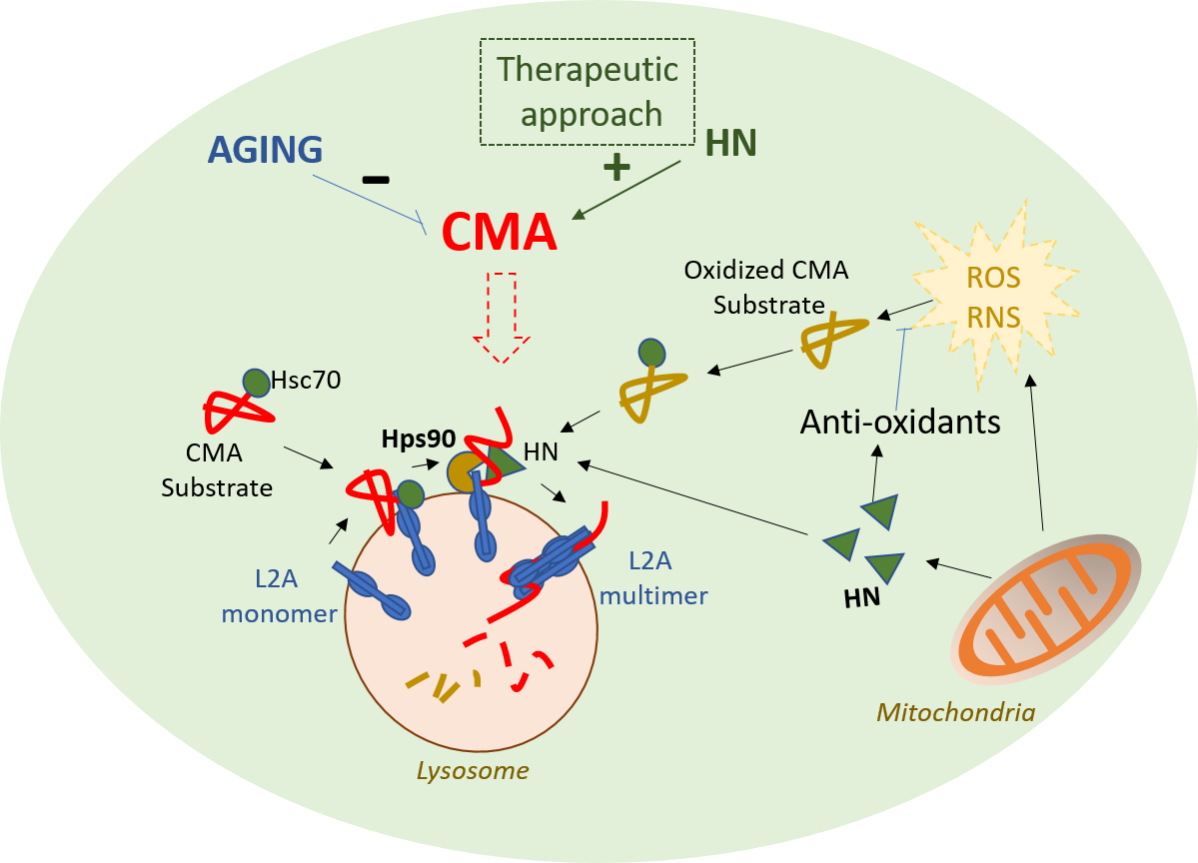
Positive effects of HN against oxidative stress through the activation of antioxidants and Chaperone-mediated autophagy (CMA) Proposed model of the stimulatory effect of HN on CMA, where HN interacts with HSP90 at the cytosolic side to enhance CMA activity under basal and stress condition (oxidative stress) could be used as a therapeutic tool in some age-related diseases where CMA activity is decreased. Abbreviations: HN: humanin, L2A: lysosome-associated membrane protein type 2A, ROS: reactive oxygen species, RNS: reactive nitrogen species.

Our study provided the first evidence that regulatory signals from mitochondria can control CMA. We propose that while generating ROS from metabolism, mitochondria simultaneously initiates signals such as HN to eliminate ROS by increasing antioxidant enzyme activities, and decrease oxidative insults by activating CMA, and that perturbations in this process could cause accumulation of oxidative damage leading to cell death and human diseases. It is interesting to note that HN and CMA both decline with age [3, 5] and that genetic correction of the CMA defect in livers from old mice was effective in improving hepatic homeostasis, conferring higher resistance to stress and improved organ function [6]. We propose that interventions aimed to enhance mitochondrial peptide HN levels could have a similar effect, and protect against oxidative stress by enhancing removal of oxidative-damage proteins through CMA (Figure 1). Whether this is a unique function of HN, or is shared by other mitochondria-encoded peptides such as small humanin like peptides (SHLPS) requires future investigation [7]. Efforts should be directed to testing a possible protective effect of HN in age-related diseases with a primary defect on CMA such as Parkinson’s disease.
